# Right ventricular dysfunction as an echocardiographic prognostic factor in hemodynamically stable patients with acute pulmonary embolism: a meta-analysis

**DOI:** 10.1186/1471-2261-14-64

**Published:** 2014-05-06

**Authors:** Jae Hyung Cho, Gurusaravanan Kutti Sridharan, Seon Ha Kim, Roop Kaw, Triveni Abburi, Affan Irfan, Abraham G Kocheril

**Affiliations:** 1 Department of Hospital Medicine, Cleveland Clinic, OH, 9500 Euclid Avenue, M2-Annex, Cleveland, OH 44195, USA; 2 Department of Internal Medicine, College of Medicine, University of Illinois at Urbana-Champaign, Champaign, IL, USA; 3 Department of Nursing, Dankook University, Cheonan, Republic of Korea; 4 Departments of Hospital Medicine and Outcomes Research Anesthesiology, Cleveland Clinic, Cleveland, OH, USA; 5 Department of Cardiology, College of Medicine, University of Illinois at Urbana-Champaign, Champaign, IL, USA

**Keywords:** Echocardiography, Pulmonary embolism, Right ventricular dysfunction

## Abstract

**Background:**

We investigated whether right ventricular dysfunction (RVD) as assessed by echocardiogram can be used as a prognostic factor in hemodynamically stable patients with acute pulmonary embolism (PE). Short-term mortality has been investigated only in small studies and the results have been controversial.

**Methods:**

A PubMed search was conducted using two keywords, “pulmonary embolism” and “echocardiogram”, for articles published between January 1st 1998 and December 31st 2011. Out of 991 articles, after careful review, we found 12 articles that investigated the implications of RVD as assessed by echocardiogram in predicting short-term mortality for hemodynamically stable patients with acute PE. We conducted a meta-analysis of these data to identify whether the presence of RVD increased short-term mortality.

**Results:**

Among 3283 hemodynamically stable patients with acute PE, 1223 patients (37.3%) had RVD, as assessed by echocardiogram, while 2060 patients (62.7%) had normal right ventricular function. Short-term mortality was reported in 167 (13.7%) out of 1223 patients with RVD and in 134 (6.5%) out of 2060 patients without RVD. Hemodynamically stable patients with acute PE who had RVD as assessed by echocardiogram had a 2.29-fold increase in short-term mortality (odds ratio 2.29, 95% confidence interval 1.61-3.26) compared with patients without RVD.

**Conclusions:**

In hemodynamically stable patients with acute PE, RVD as assessed by echocardiogram increases short-term mortality by 2.29 times. Consideration should be given to obtaining echocardiogram to identify high-risk patients even if they are hemodynamically stable.

## Background

Acute pulmonary embolism (PE), commonly originating from deep venous thrombosis (DVT), is a potentially life-threatening condition. PE incidence is approximately 69 per 100,000 according to a 25-year population-based study [1]. PE can be broadly classified as either massive or submassive. Patients with massive PE usually present with hemodynamic instability and are treated with either thrombolytic therapy or pulmonary embolectomy, while patients with submassive PE are generally hemodynamically stable and can be treated with anticoagulation alone. Recent evidence suggests that in selected low-risk patients with acute PE, outpatient care can be as safe and effective as inpatient care [2].

Untreated PE patients reportedly have a 30% mortality rate, whereas treated PE patients have an 8% mortality rate [3]. PE can cause right ventricular dilatation and dysfunction because of increased right ventricular afterload, leading to right ventricular failure and subsequently death. Echocardiogram is essential in ruling out intracardiac thrombus and has been widely used to assess right ventricular dysfunction (RVD). Few studies have investigated the prognostic implications of RVD as assessed by echocardiogram in hemodynamically stable patients with acute PE and the results have been controversial. There have been two previous meta-analyses regarding the assessment of RVD by echocardiogram in hemodynamically stable patients with acute PE that revealed increased short-term mortality [4,5]. However, the clinical impact of RVD as assessed by echocardiogram is still controversial and current venous thromboembolism guidelines do not recommend the routine echocardiogram to assess right ventricular function in hemodynamically stable patients with acute PE [6]. Hence, we conducted a new meta-analysis to examine the implication of RVD as assessed by echocardiogram in hemodynamically stable patients with acute PE.

## Methods

### Study objective

Few studies have studied whether RVD as assessed by echocardiogram increases short-term mortality (in-hospital or 30-day mortality) in hemodynamically stable patients with acute PE, which is controversial. The objective of this meta-analysis is to assess the prognostic value of RVD determination on echocardiogram in hemodynamically stable patients with acute PE. We followed the PRISMA statements for reporting meta-analyses.

### Study outcome

The study outcome of our meta-analysis was short-term mortality, which included in-hospital and 30-day mortality. Studies that investigated adverse clinical events such as intubation, cardiopulmonary resuscitation or the use of vasopressors, were excluded. Studies with 14-day, 3-month or 6-month mortality were also excluded to decrease meta-analysis heterogeneity.

### Search strategy

**Figure 1:**
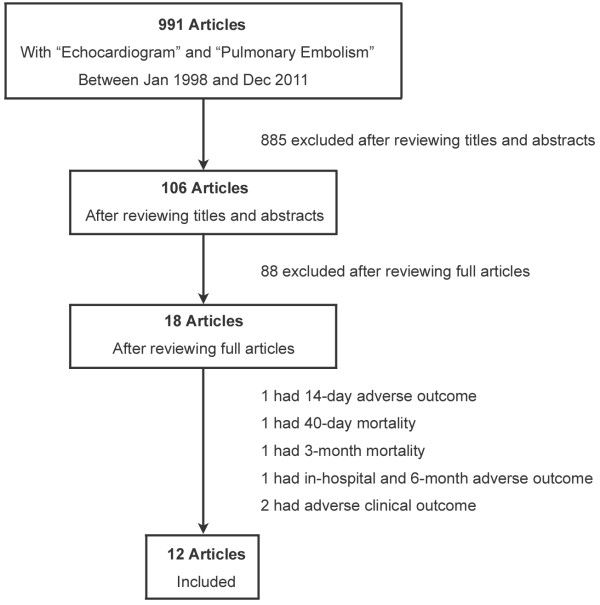
Study selection.

We searched PubMed using two keywords, “pulmonary embolism” and “echocardiogram”. Search criteria included the time frame between January 1st 1998 and December 31th 2011, humans, English, and adults older than 19 years. A total of 991 articles were included in our initial search. The articles were independently reviewed by two authors (J.H.C. and G.K.S.). The initial screening was performed by first reviewing the titles and the abstracts were reviewed if they contained text describing the prognostic value of RVD assessment. After reviewing the titles and abstracts, 106 articles were selected for full review. In total, eighteen out of 106 studies investigated the implications of RVD as assessed by echocardiogram in hemodynamically stable patients with acute PE. The rest of the eighty-eight studies were excluded for the following reasons: they were not investigational studies, they had different study objectives, they used different patient populations such as hemodynamically unstable PE or chronic PE, or they used different RVD diagnostic modalities such as CT, cardiac biomarkers or brain natriuretic peptide (BNP). In total, six studies had different clinical outcomes such as 14-day, 40-day, 3-month mortality or adverse clinical outcomes, which were excluded. Finally, twelve studies were selected for our meta-analysis (Figure 1). The disagreements between these two authors were reviewed and the two disagreeing studies were excluded because of different clinical outcomes. After meticulous review, the data were extracted independently by the two aforementioned authors using standard data extraction forms and were confirmed together.

### Eligibility criteria

We used studies that included only hemodynamically stable patients with acute PE. We excluded hemodynamically unstable patients with acute PE. Hemodynamic instability was defined as systolic blood pressure less than 90 mmHg or a systolic blood pressure drop greater than 40 mmHg from the baseline. We also excluded patients with chronic PE. We selected studies that investigated the implications of RVD assessment by echocardiogram to predict short-term mortality, which includes in-hospital or 30-day mortality. RVD can be assessed by echocardiogram, CT, cardiac biomarkers or BNP. However, we only included studies that investigated RVD using echocardiogram. We excluded studies that focused on mortality other than in-hospital or 30-day. We excluded studies that investigated adverse clinical outcomes such as intubation, cardiopulmonary resuscitation or vasopressor use.

### Statistical analysis

We performed meta-analysis using RevMan 5.0 with the Mantel-Haenszel random effects model. Cochran’s chi-square test and the I^2^ test were used to assess between-study heterogeneity. Heterogeneity was considered statistically significant at P < 0.10 and I^2^ > 50%. Pooled odds ratios were reported with 95% confidence intervals.

### Subgroup analyses

Subgroup analyses were performed according to the outcome measure (in-hospital mortality vs. 30-day mortality) and the study design (prospective vs. retrospective).

## Results

### Study selection

A total of 991 articles were included in our initial search. After a thorough review of titles and abstracts, 106 journals were selected as preliminary candidates. After reviewing full articles, 12 studies (n = 3283) were selected for data extraction and meta-analysis.

### Study characteristics

A total of 3283 patients were included in our meta-analysis. Individual characteristics of the included studies are shown in Table 1. As a study outcome, nine studies measured in-hospital mortality while three studies investigated 30-day mortality. In total, seven studies were prospective whereas five studies were retrospective. The qualitative findings of included studies using Newcastle-Ottawa quality assessment scale are described in Table 2.

**Table 1:** Study characteristics

	**Author (Journal)**	**Year**	**Study design**	**Patients number**	**Diagnostic nodality**	**RVD Definition**	**Follow-up**	**RVD (+)**		**RVD (-)**	
								**Death**	**Total**	**Death**	**Total**
1	Grifoni et al. [7] (Circulation)	2000	Prospective	162	CT, V/Q scan or pulmonary angiography	RVEDD>30mm, RV/LV>1, PSWM, or PH	In-hospital mortality	4	65	3	97
2	Yalamanchili et al. [8] (Am J Cardiol)	2004	Retrospective	91	CT	RVEDD>30mm, or RVHK and PSWM	In-hospital mortality	4	25	8	66
3	Kucher et al. [9] (Arch Intern Med)	2005	Retrospective	1035	Necropsy, V/Q scan, pulmonary angiography or high suspicion in the setting of DVT	RVHK	30-day mortality	65	405	59	630
4	Sukhija et al. [10] (Am J Cardiol)	2005	Retrospective	190	CT	Right ventricular dilatation (RV>LV, or RV>4.5cm)	In-hospital mortality	21	64	6	126
5	Grifoni et al. [11] (Arch Intern Med)	2006	Prospective	301	CT, V/Q scan or pulmonary angiography	RVEDD>30mm, RV/LV>1, PSWM, or PH	In-hospital mortality	24	146	21	155
6	Pieralli et al. [12] (Am J Cardiol)	2006	Prospective	61	CT or pulmonary angiography	RVEDD>30mm, RV/LV>1, RVHK, PSWM, or PH	In-hospital mortality	4	35	0	26
7	Jimenez et al. [13] (Arch Bronchoneumol)	2007	Prospective	214	CT or V/Q scan	RVEDD>30mm, RV>LV, or RVHK	30-day mortality	4	86	3	128
8	Logeart et al. [14] (Intensive Care Med)	2007	Prospective	67	CT or V/Q scan	Two or more of RV/LV>0.7, RVHK, IVC>10mm during inspiration, IVC bulging, TRV>2.7m/s	In-hospital mortality	1	36	0	31
9	Palmieri et al. [15] (Intern Emerg Med)	2008	Prospective	89	CT	RV/LV>0.9, PSWM, or depressed RV systolic function	In-hospital mortality	8	48	4	41
10	Gallotta et al. [16] (Int J Cardio)	2008	Prospective	90	CT	PSWM, RV diameter>15mm/m^2^, or low RV systolic function	In-hospital mortality	10	65	2	25
11	Stein et al. [17] (Am J Cardio)	2011	Retrospective	392	CT or V/Q scan	Quantitative in 325 patients, or RV/LV>1 in 67 patients	In-hospital mortality	4	264	8	128
12	Jimenez et al. [18] (Thorax)	2011	Retrospective	591	CT, V/Q scan or high suspicion in the setting of DVT	RVEDD>30mm, RV>LV, RVHK, or TRV>2.6m/s	30-day mortality	14	120	24	471

RVEDD (Right ventricular end-diastolic diameter in precordial view), RV/LV (Right ventricular/left ventricular end-diastolic diameter in 4-chamber view), PSWM (Paradoxical septal wall motion), PH (Pulmonary hypertension), RVHK (Right ventricular hypokinesia), IVC (Inferior vena cava), TRV (Tricuspid regurgitation velocity).

**Table 2:** Qualitative findings (Newcastle-Ottawa Quality Assessment Scale)

		**Selection**				**Comparability**	**Exposure**			**Total score**
	**Author (Journal)**	**Is the case definition adequate?**	**Representativeness of the cases**	**Selection of controls**	**Definition of controls**	**Comparability of cases and controls (age)**	**Ascertainment of exposure**	**Same method of ascertainment of cases and controls**	**Non-response rate**	
1	Grifoni et al. [7] (Circulation)	1	1	1	1	1	1	1	1	8
2	Yalamanchili et al. [8] (Am J Cardiol)	1	1	1	1	0	1	1	1	7
3	Kucher et al. [9] (Arch Intern Med)	0	1	1	1	1	1	1	1	7
4	Sukhija et al. [10] (Am J Cardiol)	0	1	1	1	0	1	1	1	6
5	Grifoni et al. [11] (Arch Intern Med)	1	1	1	1	1	1	1	1	8
6	Pieralli et al. [12] (Am J Cardiol)	1	1	1	1	1	1	1	1	8
7	Jimenez et al. [13] (Arch Bronchoneumol)	1	1	1	1	1	1	1	1	8
8	Logeart et al. [14] (Intensive Care Med)	1	1	1	1	1	1	1	1	8
9	Palmieri et al. [15] (Intern Emerg Med)	1	1	1	1	0	1	1	1	7
10	Gallotta et al. [16] (Int J Cardio)	1	1	1	1	0	1	1	1	7
11	Stein et al. [17] (Am J Cardio)	0	1	1	1	0	1	1	1	6
12	Jimenez et al. [18] (Thorax)	1	1	1	1	1	1	1	1	8

### Right ventricular dysfunction as assessed by echocardiogram

The American Society of Echocardiography published “Guidelines for the Echocardiographic Assessment of the Right Heart in Adults” in 2010 [19]. However, because most of our studies were performed before this guideline was published, the definition of RVD as assessed by echocardiogram differed from study to study. The echocardiographic criteria of RVD in each study are described in Table 1. The most commonly used criteria were: right ventricular end-diastolic diameter > 30 mm, right ventricular/left ventricular end-diastolic diameter > 1 or right ventricular hypokinesia. Other criteria included paradoxical septal wall motion, pulmonary hypertension and severe tricuspid regurgitation.

### Right ventricular dysfunction predicting in-hospital or 30-day mortality

**Figure 2:**
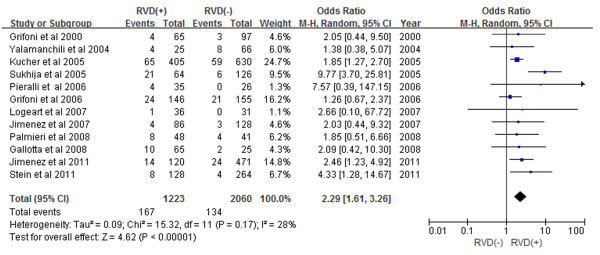
Meta-analysis of short-term mortality (In-Hospital or 30-Day Mortality).

Among 3283 hemodynamically stable patients with acute PE, 1223 patients (37.3%) had RVD as assessed by echocardiogram while 2060 patients (62.7%) had normal right ventricular function. Short-term mortality was reported in 167 (13.7%) out of 1223 patients with RVD and in 134 (6.5%) out of 2060 patients without RVD. Hemodynamically stable patients with acute PE who had RVD as assessed by echocardiogram had a 2.29-fold increase in short-term mortality (Figure 2) (odds ratio (OR) 2.29, 95% confidence interval (CI) 1.61-3.26) compared with patients without RVD.

### Subgroup analyses

**Figure 3:**
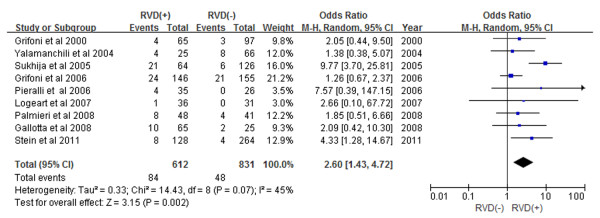
Meta-analysis of in-hospital mortality.

**Figure 4:**
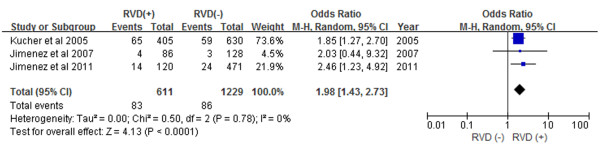
Meta-analysis of 30-day mortality.

**Figure 5:**
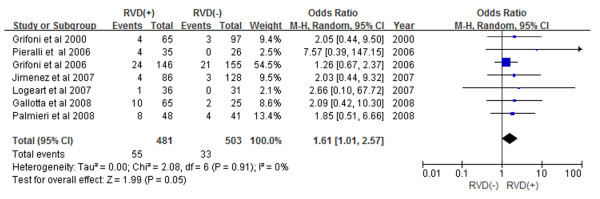
Meta-analysis of prospective studies.

**Figure 6:**
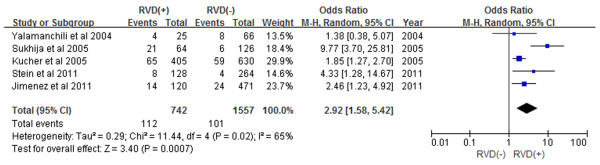
Meta-analysis of retrospective studies.

Subgroup analyses demonstrated that patients with RVD as assessed by echocardiogram had a 2.60-fold increase in in-hospital mortality (Figure 3) and a 1.98-fold increase in 30-day mortality (Figure 4) (OR 2.60, 95% CI 1.43-4.72, and OR 1.98, 95% CI 1.43-2.73, respectively). Prospective study subgroup analysis revealed that patients with RVD as assessed by echocardiogram had a 1.61-fold increase in short-term mortality (Figure 5) while retrospective studies demonstrated a 2.92-fold increase in short-term mortality (Figure 6) (OR 1.61, 95% CI 1.01-2.57, and OR 2.92, 95% CI 1.58-5.42, respectively).

### Risk of bias

To decrease the risk of bias, two authors independently assessed the validity of the included studies at the outcome level. To increase homogeneity, we only included studies with clinical outcomes of either in-hospital or 30-day mortality. There might be a publication bias despite meticulous database searches because small negative studies or positive studies without statistical significance might not get published. To minimize selection bias we used very clear inclusion and exclusion criteria.

## Discussion

RVD has long been thought to be a cause of death in patients with acute PE. Right ventricular function can be assessed using echocardiogram, CT, cardiac biomarkers or BNP. Several studies investigated the implication of elevated cardiac biomarkers or BNP levels and concluded that patients with elevated levels have an increased mortality rate [4,5,20]. Because patients with congestive heart failure, chronic kidney disease, stroke or other pulmonary disease may also have elevated baseline cardiac biomarker or BNP levels, there are limitations to assess right ventricular function. However, an echocardiogram is more specific to assess right ventricular function because we can directly image the right ventricle with the echocardiogram, which is not influenced by other co-morbidities.

Recent studies suggest that certain patients with low-risk profiles can be managed safely at home without hospitalization [2]. High-risk profiles include old age, congestive heart failure, chronic lung disease, pulse > 110 bpm, systolic blood pressure < 100 mmHg, respiratory rate > 30 breaths per min or arterial oxygen saturation < 90% according to the pulmonary embolism severity index [21,22]. Our meta-analysis indicated that patients with RVD as assessed by echocardiogram should be regarded as high-risk because RVD increases short-term mortality. However, the current guidelines of venous thromboembolism do not recommend the routine use of cardiac biomarkers, BNP, CT or echocardiogram to assess right ventricular function in hemodynamically stable patients because it will not change patient management [6]. The positive predictive value of mortality on the basis of echocardiographic evidence of RVD in hemodynamically stable patients has generally been reported to be in the 5 to 8% range [7,13]. We suggest consideration be given to obtaining echocardiogram with special emphasis on right ventricular wall motion in normotensive patients with acute PE, especially when they have dyspnea, tachypnea or tachycardia.

The use of RVD as assessed by echocardiogram as a prognostic factor in acute PE has been controversial. As described in our included studies, eight out of twelve studies demonstrated non-significant results in predicting mortality [7,8,11-16]. Only four studies revealed a statistically significantly increased mortality rate [9,10,17,18]. There have been two previous meta-analyses regarding RVD assessment by echocardiogram. Sanchez et al. included five studies and Coutance et al. included eight studies [4,5]. However, the clinical outcome of these meta-analyses had wide variations from in-hospital mortality to 14-, 30- or 40-day mortality [4,5]. Our meta-analysis included 12 studies with uniform clinical outcomes of either in-hospital or 30-day mortality. Short-term mortality, which includes in-hospital or 30-day mortality, was increased 2.29-fold in patients with RVD as assessed by echocardiogram. Subgroup analyses revealed that patients with RVD as assessed by echocardiogram have a 2.60-fold increase in in-hospital mortality and a 1.98-fold increase in 30-day mortality. None of these meta-analyses demonstrated significant heterogeneity. Although treatment of RVD without hemodynamic instability among patients with PE was not within the scope of our meta-analysis, several studies have addressed this issue [23-25]. The most recent study randomized patients with intermediate-risk PE (as defined by RVD on echocardiogram or spiral CT and myocardial injury as defined by troponin elevation) to thrombolytic agent (tenecteplase) vs. intravenous heparin [25]. Decreased composite of hemodynamic instability and death (2.6%) but higher risk of hemorrhagic stroke (2.0%) and major extracranial hemorrhage (6.3%) were noted in the group treated with tenecteplase, thus advising cautious use of thrombolytic agent among hemodynamically stable patients with RVD [25].

There are several limitations to our meta-analysis. First, we used only one database for the study selection. PubMed is the major search engine for medical literature; however, we could increase the power of our meta-analysis by including other search engines such as Cochrane or Embase. Even using only with PubMed, we found significant studies with adequate homogeneity. Second, the definition of RVD as assessed by echocardiogram differed among the studies (Table 1). In particular, in the study by Sukhija et al. it is possible that too stringent criteria for RVD (RV > LV, or RV > 4.5 cm) were used which may have contributed to a very high OR for short-term mortality [10]. In light of such high short-term mortality, it is also possible that even though the patients in this study were classified as hemodynamically stable, some of them may really have had some clinical clue or deterioration which was missed. Although the American Society of Echocardiography published a guideline of assessing the right heart in an adult population in 2010, most of the studies were performed before the publication of this guideline [19]. In the future, the homogeneity can be increased and subgroup analysis of different echocardiographic RVD criteria can be performed if we have sufficient studies that followed this guideline. Third, it is hard to assess whether RVD is an acute finding secondary to PE or a chronic condition because of other co-morbidities because most of these patients do not have a baseline echocardiogram to check right ventricular function. Differentiation of acute versus chronic RVD would also be difficult solely on echocardiographic findings. Because it is not practical to obtain a baseline echocardiogram before PE development, this assessment will remain a future research area. Lastly, some studies in the meta-analysis lacked stringent exclusion criteria and it is possible that hemodynamic instability was under-reported.

## Conclusions

In hemodynamically stable patients with acute PE, RVD as assessed by echocardiogram increases short-term mortality by 2.29 times. Consideration should be given to obtaining echocardiogram to identify high-risk patients even if they are hemodynamically stable.

## Abbreviations

PE: Pulmonary embolism; DVT: Deep vein thrombosis; RVD: Right ventricular dysfunction; BNP: Brain natriuretic peptide; RVEDD: Right ventricular end-diastolic diameter in precordial view; RV/LV: Right ventricular/Left ventricular end-diastolic diameter in 4-chamber view; PSWM: Paradoxical septal wall motion; PH: pulmonary hypertension; RVHK: Right ventricular hypokinesia; IVC: Inferior vena cava; TRV: Tricuspid regurgitation velocity; OR: Odds ratio; CI: Confidence interval.

## Competing interests

The authors declare that they have no competing interests.

## Authors’ contributions

JHC, GKS, SHK, RK, TA, AI and AGK contributed to the writing of the manuscript. All authors reviewed and approved the final version of the manuscript.

## Pre-publication history

The pre-publication history for this paper can be accessed here:

http://www.biomedcentral.com/1471-2261/14/64/prepub
